# Genome Analysis of a Potential Novel *Vibrio* Species Secreting pH- and Thermo-Stable Alginate Lyase and Its Application in Producing Alginate Oligosaccharides

**DOI:** 10.3390/md22090414

**Published:** 2024-09-10

**Authors:** Ke Bao, Miao Yang, Qianhuan Sun, Kaishan Zhang, Huiqin Huang

**Affiliations:** 1 Institute of Tropical Bioscience and Biotechnology, Hainan Institute for Tropical Agricultural Resources, Chinese Academy of Tropical Agricultural Sciences, Haikou 571101, China; 17326063163@163.com (K.B.); 15840046287@163.com (M.Y.); 2 Hangzhou Watson Biotechnology Co., Ltd., Hangzhou 311400, China; sunqianhuan@watsonbiotech.com; 3 College of Life Science and Technology, Huazhong Agricultural University, Wuhan 430070, China

**Keywords:** alginate lyase, *Vibrio* sp. HB236076, genome, oligosaccharide, antimicrobial activity

## Abstract

Alginate lyase is an attractive biocatalyst that can specifically degrade alginate to produce oligosaccharides, showing great potential for industrial and medicinal applications. Herein, an alginate-degrading strain HB236076 was isolated from *Sargassum* sp. in Qionghai, Hainan, China. The low 16S rRNA gene sequence identity (<98.4%), ANI value (<71.9%), and dDDH value (<23.9%) clearly indicated that the isolate represented a potential novel species of the genus *Vibrio*. The genome contained two chromosomes with lengths of 3,007,948 bp and 874,895 bp, respectively, totaling 3,882,843 bp with a G+C content of 46.5%. Among 3482 genes, 3332 protein-coding genes, 116 tRNA, and 34 rRNA sequences were predicted. Analysis of the amino acid sequences showed that the strain encoded 73 carbohydrate-active enzymes (CAZymes), predicting seven PL7 (Alg1–7) and two PL17 family (Alg8, 9) alginate lyases. The extracellular alginate lyase from strain HB236076 showed the maximum activity at 50 °C and pH 7.0, with over 90% activity measured in the range of 30–60 °C and pH 6.0–10.0, exhibiting a wide range of temperature and pH activities. The enzyme also remained at more than 90% of the original activity at a wide pH range (3.0–9.0) and temperature below 50 °C for more than 2 h, demonstrating significant thermal and pH stabilities. Fe^2+^ had a good promoting effect on the alginate lyase activity at 10 mM, increasing by 3.5 times. Thin layer chromatography (TLC) and electrospray ionization mass spectrometry (ESI-MS) analyses suggested that alginate lyase in fermentation broth could catalyze sodium alginate to produce disaccharides and trisaccharides, which showed antimicrobial activity against *Shigella dysenteriae*, *Aeromonas hydrophila*, *Staphylococcus aureus*, *Streptococcus agalactiae*, and *Escherichia coli*. This research provided extended insights into the production mechanism of alginate lyase from *Vibrio* sp. HB236076, which was beneficial for further application in the preparation of pH-stable and thermo-stable alginate lyase and alginate oligosaccharides.

## 1. Introduction

Alginate is a class of polysaccharides widely distributed in brown seaweeds such as *Laminaria* and *Sargassum*, with a content of approximately 30–60% of their dry weight [1]. As a water-soluble acidic polysaccharide, it is constituted by two isomer residues, α-l-guluronic acid (G) and β-d-mannuronic acid (M). These two residues are connected by 1, 4-glycosidic bonds to contribute three different blocks, namely polyM, polyG, and polyMG blocks [2]. Alginate, with high viscosity and gelling properties, has enormous potential for application in the food, cosmetics, and pharmaceutical industries [3]. Meanwhile, the low water solubility and high solution viscosity limit its application when high concentrations are needed, especially in food products.

Alginate oligosaccharides (AOS) are molecules formed by the decomposition of alginate with a degree of polymerization (DP) of 2 to 25. They have shorter chain lengths and thus improve water solubility compared to higher molecular-weight alginates with the same monomers. Many pathways can be used to degrade alginate into AOS with low DP, including physical, chemical, and enzymatic degradation. Compared with physical and chemical methods, the enzymatic methods have the advantages of high efficiency, specificity, safety, and decreased byproducts [4]. Alginate lyases play a key role in alginate degradation through an enzymatic route and can be used for the directional preparation of AOS with a specific monosaccharide and DP [5]. Owing to a double bond between C4 and C5 at the non-reducing end, AOS exhibit higher biological activities [6]. Recently, AOS have gained growing attention because of their important biological functions such as antimicrobial, antioxidant, prebiotic, antitumor, anticoagulant, immunomodulatory, and plant root growth-promoting activities [6,7,8,9,10,11]. Due to these characteristics, AOS have broad application potential in the agricultural, food, and pharmaceutical industries [12].

Alginate lyases can depolymerize alginate into oligosaccharides or monosaccharides by cleaving glycosidic bonds through a β-elimination reaction [13]. According to substrate specificity, alginate lyases can be divided into polyG-specific lyases (EC 4.2.2.11), polyM-specific lyases (EC 4.2.2.3), and polyMG-specific lyases (EC 4.2.2), which can degrade polyG, polyM, and polyMG blocks of alginate, respectively [14]. In the Carbohydrate-active enzymes (CAZymes) database (http://www.cazy.org/ (accessed on 15 June 2024)), alginate lyases are categorized into 15 polysaccharide lyase (PL) families (PL5, 6, 7, 14, 15, 17, 18, 20, 31, 32, 34, 36, 38, 39, and 41) based on their amino acid sequences, which imply significant structural diversity of the catalytic sites [15]. Alginate lyases come from a wide range of sources, including marine mollusks, bacteria, fungi, and marine brown alga [16]. Numerous studies have reported the ability of bacteria to degrade alginates, such as *Alteromonas* [17], *Pseudoalteromonas* [18], *Paenibacillus* [19], *Microbulbifer* [20], and *Zobellia* [21]. As is well known, members of the genus *Vibrio* can also degrade alginate due to their alginolytic systems, which include diverse alginate lyases. A variety of alginate lyases have been explored from *Vibrio* species, for instance, *V. harveyi* AL-128 [22], *V. splendidus* 12B01 [23], *V. xiamenensis* QY104 [24], *Vibrio* sp. dhg [25], and *Vibrio* sp. W13 [26].

In this study, an alginate-degrading strain HB236076 was isolated from *Sargassum* sp. in Qionghai, Hainan, China. Based on the 16S rRNA gene and genome sequence analyses, the strain was assumed to represent a potential novel species of the genus *Vibrio*. The general characteristics of its genome sequence were reported, and genome annotation showed that it contained abundant alginate lyases. In addition, we further investigated the properties of alginate lyase in culture supernatant and the AOS produced by enzyme degradation.

## 2. Results

### 2.1. Screening of Strain HB236076

The screening results obtained by the agar plate method showed that strain HB236076 from *Sargassum* sp. exhibited significant alginate lyase activity. Under the action of 1 M CaCl_2_, a white ring produced by gelation reaction on the plate showed that alginate lyase was secreted (Figure S1).

### 2.2. Genome Specifics

**Figure 1:**
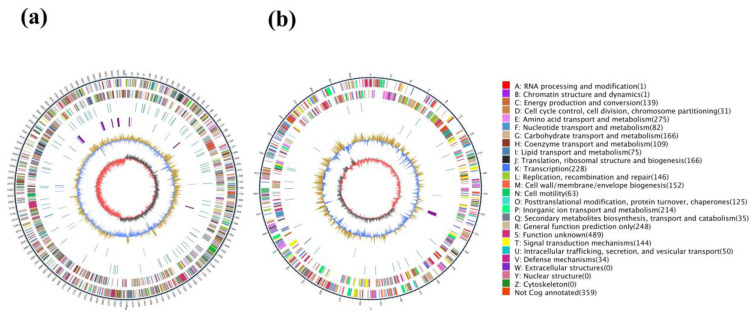
Circos maps of the two chromosome genomes of strain HB236076. (**a**) Chromosome 1; (**b**) chromosome 2. The maps were divided into 7 circles from outside to inside, namely, markers of genome size (5 kb per scale), genes on the positive strand, genes on the negative strand, repetitive sequences, genes of tRNA (blue) and rRNA (purple), GC content (buff: above mean; blue: below mean), and GC-skew (dark gray: G higher than the C; red: G lower than the C).

The complete genome of strain HB236076 was determined, and two circular chromosomes were obtained, with the NCBI GenBank accession numbers CP162601 and CP162602. A total of 65,029 clean reads with an average read length of 8717 bp, totaling 566.9 Mb, were analyzed, giving 146× coverage depth. Strain HB236076 presented two chromosome genomes of 3,007,948 bp and 874,895 bp, totaling 3,882,843 bp, with a G+C content of 46.5%. A total of 3482 genes were predicted, including 3332 protein-coding genes, 116 tRNA, and 34 rRNA (12, 11, 11 for 5S, 16S, and 23S rRNAs, respectively) sequences. The general features of strain HB236076 genome are shown in Figure 1 and Table S1.

The gene functions were classified with KEEG databases, and a total of 2123 proteins were annotated. ABC transporters with a total of 170 genes accounted for 8.0% of the annotated genes and comprised the highest proportion in the KEGG pathway, followed by biosynthesis of amino acids (5.2%), two-component system (4.9%), carbon metabolism (3.9%), purine metabolism (3.7%), and pyrimidine metabolism (2.8%). Bioinformatics analysis using antiSMASH predicted four antibiotic resistance proteins, namely fluoroquinolone, penam, macrolide, and tetracycline antibiotics.

### 2.3. Identification of Strain HB236076

**Figure 2:**
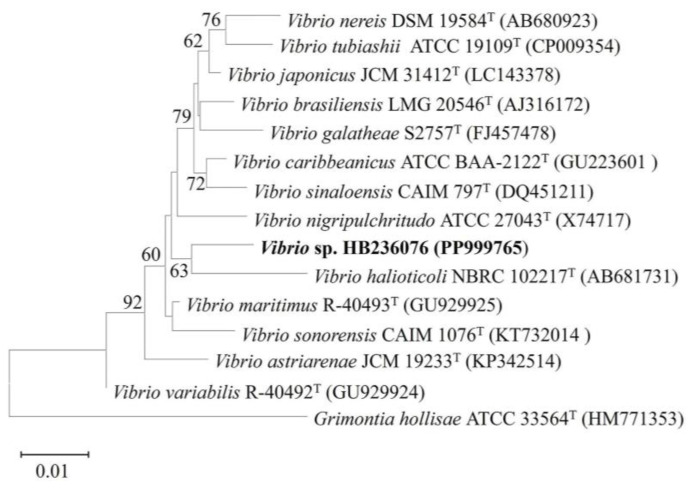
Neighbor-joining phylogenetic tree based on 16S rRNA gene sequences between strain HB236076 and related species of the genus *Vibrio* by comparison of 1478 nucleotides. Bootstrap values (1000 replicates) are shown as percentages at each node for values; only values > 50% are shown. *Grimontia hollisae* ATCC 33564^T^ was used as an outgroup. The scale bar represents 0.01 nucleotide substitutions per position.

After culturing on MA at 30 °C for 2 d, colonies of strain HB236076 were beige, circular, regular, smooth, and 1 mm in diameter. A nearly complete 16S rRNA gene sequence (1474 bp) by PCR amplification was obtained, and the complete sequence (1552 bp) was determined from the genome with GenBank No. PP999765. Phylogenetic analysis indicated that the isolate belonged to the genus *Vibrio*. The closest phylogenetically related species were *V. maritimus* R-40493^T^, *V. variabilis* R-40492^T^, *V. japonicus* JCM 31412^T^, and *V. sinaloensis* CAIM 797^T^, with 98.4, 98.1, 98.0, and 98.0% similarities, respectively. The other 16S rRNA gene sequence similarities were under 98.0%. The phylogenetic tree reconstructed using the neighbor-joining method showed that the strain clustered together with *V. halioticoli* NBRC 102217^T^ (with 16S rRNA gene similarity 97.4%), significantly supported by a bootstrap value of 63%, which indicated that strain HB236076 had the closest phylogenetic affinity to *V. halioticoli* (Figure 2). The low levels of sequence similarities are much lower than the 98.65% threshold for recognizing a novel species, indicating that this strain may represent a novel species of the genus *Vibrio* [27]. At the genomic level, strain HB236076 exhibited ANI values of 70.8–71.9% and dDDH values (the recommended results from formula 2) of 22.3–23.9% with the upper five closest relatives (Table S2), which were lower than the threshold values of the species boundary (ANI 94–96% and dDDH 70%) [28,29]. Hence, clearly, the results of the phylogenetic and genotypic analysis clearly indicated that strain HB236076 represented a potential novel species of the genus *Vibrio*. It was temporarily named *Vibrio* sp. HB236076 in this article.

Members of the genus *Vibrio*, widely distributed in marine environments, are an important group for alginate degradation, from which a large number of alginate lyases have been elucidated. Algb from marine bacterium *Vibrio* sp. W13 was identified as a novel endolytic alginate lyase that produced oligosaccharides with DP2–5 in an endolytic manner [26]. AlyH1 from *V. furnissii* H1 was a novel and potential candidate in the application of alginate oligosaccharides production with low DP [30]. AlyC8 from *Vibrio* sp. C42 was a novel alginate lyase with two functional catalytic domains that were synergistic in alginate degradation [31]. Herein, *Vibrio* sp. HB236076, derived from *Sargassum* sp. in the South China Sea, provided a new source for alginate lyase.

### 2.4. Genetic Basis of Alginate Degradation

In order to elucidate the related polysaccharide-degrading enzymes, the CAZymes were predicted using the dbCAN and InterPro databases. Strain HB236076 had 73 CAZymes, including 25 glycoside hydrolases (GHs), 28 glycosyl transferases (GTs), 11 polysaccharide lyases (PLs), 4 carbohydrate esterases (CEs), 3 carbohydrate-binding modules (CBMs), and 2 auxiliary activities (AAs). As shown in Table 1, nine of the putative PLs were predicted to encode alginate lyases, named Alg1–9, which indicated that *Vibrio* sp. HB236076 had powerful alginate-degrading ability. The open reading frames (ORFs) consisted of 840, 1959, 1032, 858, 747, 1029, 909, 2163, and 2163 bp nucleotides and encoded 279, 652, 343, 285, 248, 342, 302, 720, and 720 amino acids, respectively. The enzymes of Alg1–7 belonged to the PL7 family, and the other two enzymes belonged to the PL17 family. The sequence alignment results showed that Alg7 had the lowest identity among all the nine alginate lyases, with the highest sequence identity (47.9%) with polysaccharide lyase PL7 (WP_318074641.1) from unclassified *Vibrio*. Generally speaking, bacteria can secrete a series of lyases or glycosidases to depolymerize polysaccharides and produce oligosaccharides or monosaccharides that can be utilized by cells. CAZymes typically account for no more than about 2% in most bacterial genomes and seldom exceed 5% in the genomes of bacteria that specialize in carbohydrate degradation [32]. Overall, the number of CAZymes produced by the strain is not significant, accounting for 2.2%, but it has abundant alginate lyases, accounting for 12.3% of the CAZymes, indicating that the isolate has a strong ability to degrade alginate.

**Figure 3:**
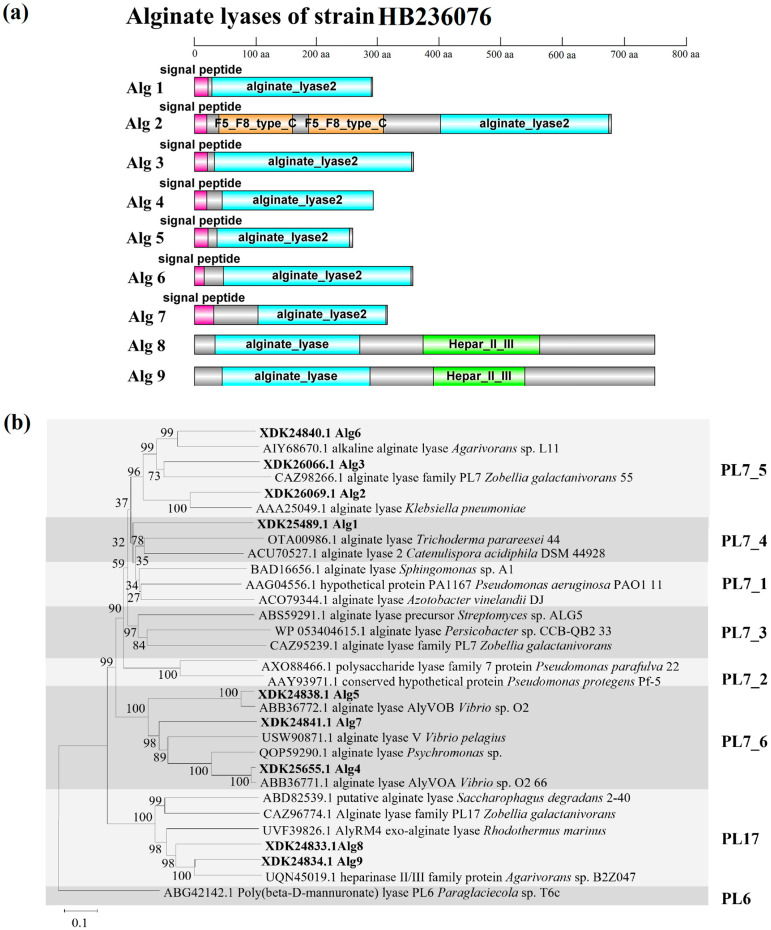
(**a**) Domain architectures of the nine alginate lyases of strain HB236076. (**b**) Neighbor-joining phylogenetic tree of alginate lyases based on the predicted amino acid sequences. Bootstrap values (1000 replicates) are shown as percentages at each node for values. The scale bar represents 0.1 nucleotide substitutions per position. Putative alginate lyases of strain HB236076 are highlighted in bold. Poly (beta-D-mannuronate) lyase (ABG42142.1) from the PL6 family was used as an outgroup.

As shown in Figure 3a, Alg1, 3–7 had only one domain but had different modularity; Alg2 had three domains, namely a catalytic domain of alginate lyase and two F5_F8 type_C domains generally known as the discoidin domain [33]; Alg8 and Alg9 had two structural domains, namely alginate lyase and Hepar_II_III domains, which meant the two enzymes of PL17 family could not only degrade alginate but also had the ability to degrade heparin and heparin sulfate. The signal peptide was predicted in Alg1–7, indicating that the seven alginases might be extracellular enzymes, while Alg8 and Alg9 did not have signal peptides and might be intracellular enzymes. In the phylogenetic tree of alginate lyases, including PL7 and PL17 families (Figure 3b), Alg1–7 were clearly located in the clade of the PL7 family, while Alg8 and Alg9 formed a distinct branch of the PL17 family and clustered in the same clade. Alg1 was classified within subfamily 4 of the PL7 family; Alg2, 3, and 6 were classified within subfamily 5 of the PL7 family; Alg4, 5, and 7 were classified within subfamily 6 of the PL7 family. The clustering results were consistent with the results predicted by the CAZy database.

**Figure 4:**
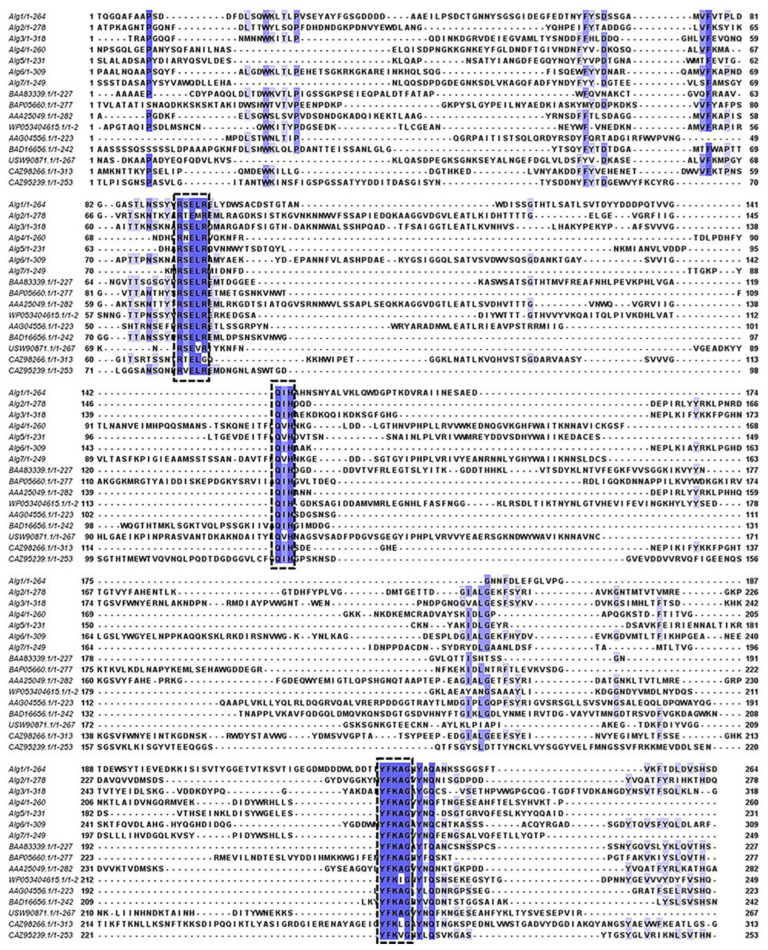
Multiple sequence alignments of Alg1–7 and nine well-characterized alginate lyases of the PL7 family. The conserved amino acid regions are marked in the black boxes.

According to the analysis of amino acid sequence, the multiple sequence alignments of Alg1–7 and nine well-characterized alginate lyases of the PL7 family are shown in Figure 4. Alg1–7 contained three highly conserved regions of the PL7 family, namely, R(S/T/V)ELR, Q(I/V)H, and YFKAG, which had an indispensable influence on the substrate binding and catalytic activity of the enzymes [18,33]. It is reported that the conserved domain of the region “QIH” means that it is more inclined to degrade polyG, while “QVH” means that it is more inclined to use polyM as the substrate [34]. The alginate lyases containing the QIH region, such as Alg2951 from *Alteromonas portus* [35] and ALY-1 from *Corynebacterium* sp. strain ALY-1 [36], degraded polyG in activity assays. Alginate lyases containing the QVH region, such as alginate lyase AlyVOA and AlyVOB from *Vibrio* sp. O2 [37] and A9m from *Vibrio* sp. JAM-A9m [38], preferred polyM as the substrate. According to the Q(I/V)H region, Alg1–3, 6 containing QIH were predicted to be polyG-preferred alginate lyases, while Alg4, 5, 7 containing QVH might be polyM-preferred alginate lyases. It is speculated that strain HB236076 could degrade polyG, polyM, and alginate.

In addition to the enzymes responsible for degrading alginate, other polysaccharide-degrading enzymes were predicted, including two α-amylases of the GH13 family, one β-galactosidase of the GH2 family, one glucosylceramidase of the GH30 family, one peptidoglycan hydrolase of the GH73 family, and one 1,4-α-glucan-branching enzyme (GlgB) of the GH13 family (Table S3). α-Amylases are a well-known type of endo-amylases and have been found in a large number of microorganisms, such as *Bacillus amyloliquefaciens*, *Bacillus licheniformis*, *Anoxybacillus beppuensis*, and *Pyrococcus woesei* [39], which have proved to be of great value in all starch-based industries. β-Galactosidase is a glycoside hydrolase that primarily breaks down lactose into glucose and galactose in bacteria, entering the glycolysis process to provide energy and carbon sources. GlgB is responsible for the transfer of chains of α-1,4-linked glucosyl residues to other similar chains (in new α-1,6 linkages) in the biosynthesis of glycogen, that is, the degree of α-1,6 branch linkages found in polysaccharides.

### 2.5. Detection of Activity and Properties of Alginate Lyase 

The fermentation supernatant was obtained by centrifugation, the alginate lyase activity of which was 39.1 U/mL, determined by the ultraviolet absorption method. Extracellular alginate lyase was prepared from the fermentation supernatant by precipitating in saturated ammonium sulfate (80%) buffer and dialyzing in a dialysis bag. The enzyme activity of the dialysis supernatant was 320.6 U/L, which was used as a partially purified alginate lyase for enzymatic properties.

**Figure 5:**
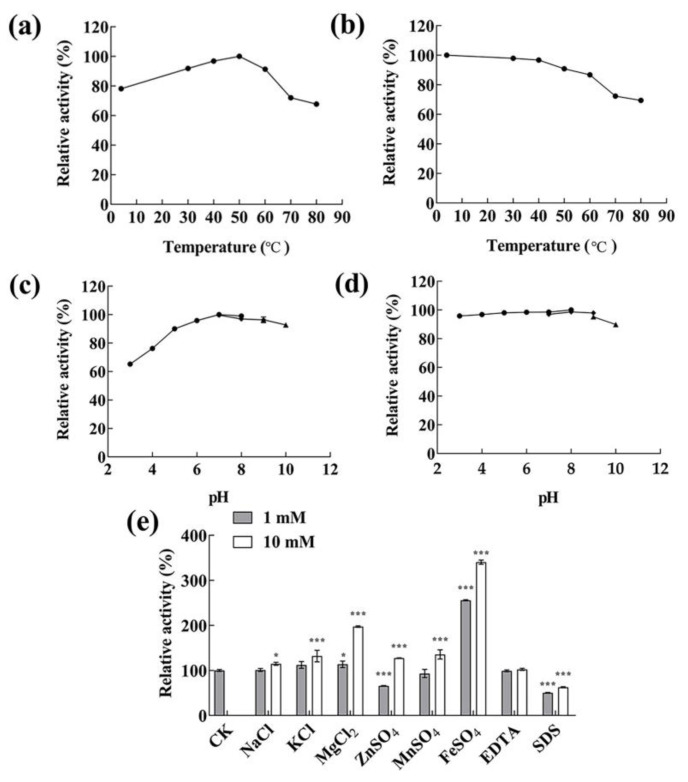
The biochemical characteristics of the extracellular alginate lyases produced by strain HB236076. (**a**) Effect of different temperatures on the alginate lyase activity assayed at 4–80 °C. (**b**) Thermal stability analysis of alginate lyase assayed at 4–80 °C for 2 h. (**c**) Effect of different pH values on the alginate lyase activity assayed at pH 3–10. (**d**) pH stability analysis of alginate lyase assayed at pH 3–10 for 24 h. (**e**) Effects of metal ions, EDTA, and SDS on the alginate lyase activity. * *p* < 0.05, *** *p* < 0.001. The highest activity was taken as 100% in Figure 5a–d, and the initial activity without additional substance was taken as 100% in Figure 5f. Data are shown as the means ± standard deviation, *n* = 3.

The effects of temperature and pH on the activity of the partially purified alginate lyase were investigated. As shown in Figure 5a, the alginate lyase exhibited maximum enzymatic activity at 50 °C. Nearly 90% of the highest activity was indicated at the temperature range of 30–60 °C, while above 70% detectable activity was observed at 4 °C and 70 °C. The thermostability of alginate lyase was measured at a temperature ranging from 4–80 °C (Figure 5b). The alginate lyases were relatively stable at temperatures below 50 °C; approximately 90% of the activity was maintained after incubation at less than 50 °C for 2 h. When the temperature rose to above 60 °C, the rate of decrease in activity accelerated, while it still maintained over 60% activity at 80 °C for 2 h. As shown in Figure 5c, the activity was the highest at pH 7.0, and nearly 90% of the maximum activity was maintained when the pH value was between 5.0 and 10.0. The activity was the most stable at pH 8.0; above 90% of the activity was maintained at pH 3.0–9.0 for 24 h (Figure 5d). The results manifested that the alginate lyases of *Vibrio* sp. HB236076 exhibited good activity under wide temperature and pH ranges and exhibited excellent thermal and pH stabilities. As shown in Figure 5e, the effects of metal ions and compounds (1 mM and 10 mM) on alginase activity were detected. At 1 mM, Fe^2+^ displayed a significant promoting effect on the enzyme activity, with 2.6 times the control group; K^+^ and Mg^2+^ displayed a weak promoting effect; Zn^2+^, Mn^2+^, and SDS showed obvious inhibitory effects. At 10 mM, Fe^2+^ and Mg^2+^ greatly increased alginate lyase activity, reaching 3.5 and 1.9 times of the control group, respectively; the other tested ions (Na^+^, K^+^, Zn^2+^, and Mn^2+^) had slightly promoted effects; SDS showed an obvious inhibiting effect. Surprisingly, 1 mM and 10 mM EDTA had no effect on the enzyme activity. It might be because the activity of the alginate lyase did not necessarily depend on the metal ions, or the binding between metal ions and enzymes was too strong, making EDTA difficult to chelate these metal ions.

In general, most alginate lyases from the PL7 family exhibited activity at a neutral temperature and pH, high activity within narrow temperature and pH ranges, and instability under high/low temperatures and acidic/alkaline conditions. For example, the AlyIH from *Isoptericola halotolerans* maintained stability at neutral pH (7.0–8.0) and temperatures below 50 °C [40], and Alg2951 from *Alteromonas portus* remained stable at pH 8.0 and temperatures below 30 °C [35]. Nevertheless, some alginate lyases from the genus *Vibrio* showed pH-stable or thermo-stable properties. Alyw202 from *Vibrio* sp. W2 exhibited the highest activity at 45 °C and more than 60% of the activity in a broad pH range of 3.0 to 10.0 [41]. Aly08 from *Vibrio* sp. SY01 remained more than 80% of its initial activity in a wide pH range (4.0–10.0) and recovered 70.8% of its initial activity following heat shock treatment for 5 min [42]. Alyw201 from *Vibrio* sp. W2 performed more than 80% of activity at 25–40 °C, and more than 70% of the activity was obtained in a broad pH range of 5.0–10.0 [43]. In this article, the alginate lyase from *Vibrio* sp. HB236076 showed maximum activity at 50 °C and pH 7.0, and over 90% of the maximum enzyme activity was measured in the range of 30–60 °C and pH 5.0–10.0, respectively. Approximately 90% of the activity remained after incubation at less than 50 °C for 2 h, or pH 6.0–10.0 for 24 h. The alginase exhibited a wide range of temperature and pH activities, as well as excellent temperature and pH stability.

### 2.6. Enzymatic Degradation of Sodium Alginate

**Figure 6:**
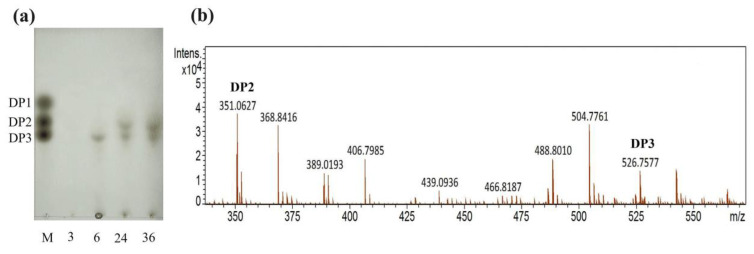
Degradation product analysis of alginate performed by alginate lyase from strain HB236076. (**a**) Degradation product analysis with TLC. Lane M, the guluronic acid sodium salt monomers, dimers, and trimers. Lane 3, 6, 24, and 36, the degradation products performed for 3, 6, 24, and 36 h, respectively. (**b**) Degradation product analysis with ESI-MS. The DP2 and DP3 peaks represent disaccharide and trisaccharide, respectively.

The partially purified alginate lyase was used to degrade polysaccharides, while TLC and ESI-MS were used to detect AOS with low DP produced by the alginate lyase. As shown in Figure 6a, no small molecule oligosaccharide appeared when incubating for 3 h. When incubated for 6 h, trisaccharides began to appear. When incubated for 24–36 h, the AOS of disaccharides and trisaccharides appeared. The oligosaccharide content at 36 h was higher than that at 24 h, and the product incubated at 36 h was used to detect by negative-ion ESI-MS (Figure 6b). The main products of the enzymatic hydrolysates were clearly distributed in disaccharides ([ΔDP2 − H]^−^ = 351.06 *m/z*) and trisaccharides ([ΔDP3 − H]^−^ = 526.76 *m/z*). The result indicated that all the extracellular alginate lyases, Alg1–7, were endo-type alginate lyases.

Current research shows that most alginate lyases exhibit polyM-specific and endo-type degradation characteristics. The majority of endo-type alginate lyases come from PL5 and PL7 families, while most exo-type alginate lyases belong to PL15 and PL17 families [44,45]. Different endo-type lyases produce different oligosaccharides, with main products typically ranging from DP2 to DP5, while exo-type lyases generally create 4-deoxy-L-erythro-5-hexoseulose uronic acid (DEH) from alginate polymers or oligosaccharides. Moreover, some lyases can perform endo- and exo-functions at the same time, such as FlAlyC of the PL6 family [46], Alg2951 of the PL7 family [35], and Alg17B of the PL17 family [47]. Up to now, most of the characterized alginate lyases of the PL7 family are endolytic enzymes, which release oligosaccharides with low DP 2–5 as the main products [2]. In this study, the alginate lyases from the fermentation supernatant specifically degrade alginate into disaccharides and trisaccharides, and no monosaccharides. It is speculated that all the extracellular PL7 alginate lyases (Alg1–7) with signal protein were endo-type enzymes and could be used to prepare oligosaccharides of DP2 and DP3. Alg8 and Alg9 of the PL17 family without signal protein were speculated to be exo-type enzymes, which were responsible for degrading oligosaccharides into monosaccharides for cellular utilization within the cell. 

### 2.7. Antimicrobial Activity of Alginate Oligosaccharides

The AOS degraded by the partially purified alginate lyase from strain HB236076 were investigated for antibacterial activity against several pathogens by the agar diffusion method. Antibacterial circles were observed around the wells in plates containing *Shigella dysenteriae*, *Aeromonas hydrophila*, *Staphylococcus aureus*, *Streptococcus agalactiae*, and *Escherichia coli,* while the AOS were 1.91 mg/mL (Figure S2). Nevertheless, no antimicrobial activity was detected when *Pseudomonas aeruginosa* and *Vibrio alginolyticus* were tested. 

Bacterial biofilms provide resistance and tolerance to host immune defense and antibiotics; therefore, disrupting biofilms is a key step in eradicating persistent bacterial infections. Alginate oligosaccharide (OligoG), consisting of 96% α-l-guluronic acid and 4% β-d-mannuronic acid isomer, possesses antibacterial and anti-biofilm properties and, thus, potentiates the activity of certain antibiotics against multidrug-resistant bacteria [48]. Many AOS have exhibited strong antibacterial activity against plant and animal pathogens. The AOS, with an average DP of 6.8, was prepared from alginate by enzymatic digestion using alginase from *Flavobacterium* sp. LXA showed significant inhibition of *Pseudomonas aeruginosa* [49]. Enzymatic AOS could also decrease *Salmonella* colonization and improve the intestinal barrier function and performance of chickens [50]. Owing to differences in degradation mode, molecular weight, G/M ratio, and spatial conformation of degradation products, AOS exhibit various biological activities, such as antitumor, antibacterial, neuroprotective, antioxidant, promoting cell proliferation, and regulating plant growth activity. It is necessary to conduct more detailed activity research on the AOS degraded by the alginate lyase. The purification, characterization, and activity test of the alginate lyases were carried out separately in our laboratory. In conclusion, the potential novel strain of *Vibrio* sp. HB236076 can be used to prepare oligosaccharides of low molecular weights with potential pharmaceutical applications.

## 3. Materials and Methods

### 3.1. Materials and Strains

Sodium alginate derived from brown seaweed was obtained from Sangon (Shanghai, China). Except for the fact that the ethanol used for purifying AOS was of chromatographic grade, all other chemicals and reagents used in this study were of analytical grade. The bacteria used as test pathogens in this study were *Staphylococcus aureus*, *Escherichia coli*, *Pseudomonas aeruginosa*, *Streptococcus agalactiae*, *Aeromonas hydrophila*, *Vibrio alginolyticus*, and *Shigella dysenteriae*, all of which were supplied from Hainan Medical University.

### 3.2. Screening of Alginate-Degrading Bacteria 

*Sargassum* samples were collected from Qionghai, Hainan, China (110°40′12′′ E, 19°18′36′′ N). A 10-gram mashed sample was diluted with 90 mL sterile aged seawater and shaken thoroughly. The suspension liquid was then serially diluted with sterile aged seawater and spread on modified 2216E agar (MA; Solarbio, Beijing, China) supplemented with 0.5% sodium alginate. The plates were monitored at 24 h intervals for 5 d at 30 °C, and colonies with different morphologies were picked up and purified. The alginate-degrading activity was preliminarily screened with the agar plate method [51]. When visible colonies were formed, 10 mL 1 M CaCl_2_ solution were poured onto the plates. After incubating at room temperature for several minutes (usually 5–10 min), strain HB236076 with gelation reaction was picked and preserved at −70 °C supplemented with 20% (*v*/*v*) glycerol. 

### 3.3. Phylogenetic Analysis Based on 16S rRNA Gene

The 16S rRNA gene sequence of strain HB236076 was amplified by PCR using two universal primers, 27F and 1492R, cloned into vector pMD 19^T^ (TaKaRa), then sequenced to determine the almost-complete sequence of the 16S rRNA gene [52]. The determined 16S rRNA gene sequence was compared with sequences of the closely related reference organisms at the EzBioCloud (https://www.ezbiocloud.net/identify, accessed on 15 June 2024) and NCBI nucleotide database (https://www.ncbi.nlm.nih.gov/, accessed on 15 June 2024). Multiple sequence alignment and analysis of the corresponding sequences of the most closely related type strains were carried out using the MEGA (molecular evolutionary genetics analysis) version 11 software package [53]. Phylogenetic trees were constructed using the neighbor-joining method, and evolutionary distances were calculated with the Kimura-2-parameter model based on 1000 replicates bootstrap analysis [54]. *Grimontia hollisae* ATCC 33564^T^ was used as an outgroup in the phylogenetic tree.

### 3.4. Genome Sequencing and Annotation

Cells grown on MA agar for 24 h were harvested to extract genomic DNA using the TIANamp Bacteria DNA Kit (Qiagen, DP302) following the manufacturer’s protocol. The quantity and quality of the DNA were checked by 0.8% agarose gel electrophoresis, NanoDrop 2000 (Thermo Scientific, Waltham, MA, USA) and Qubit version 2.0 fluorometer (Invitrogen, Carlsbad, CA, USA). The high-quality genome sequence was sequenced using the PacBio Sequel II system (Pacific Biosciences, Menlo Park, CA, USA) at Biomarker Technologies Co., Ltd. (Beijing, China). The de novo genome assembly was performed using Hifiasm v0.12. The clean data from Illumina Hiseq2500 (San Diego, CA, USA) was sequenced using Pilon v1.22 to generate one contig without a gap to verify the contaminant. G+C content was analyzed using the RAST server and the genome sequence [55]. Gene and protein-coding sequences were predicted with Prodigal v2.6.3 [56]. Transfer RNA (tRNA) and ribosomal RNA (rRNA) were predicted with tRNAscan-SE v2.0 [57] and Infernal v1.1.3 [58], respectively. The genome was annotated with the non-redundant protein (NR), gene ontology (GO), and Kyoto Encyclopedia of Genes and Genomes (KEGG) results for gene function prediction [59,60,61]. The secondary metabolite gene clusters were predicted by antiSMASH 6.0 [62].

CAZymes were annotated using HMMER software against the dbCAN database (http://bcb.unl.edu/dbCAN2, accessed on 30 July 2024) [63] and InterPro database (https://www.ebi.ac.uk/interpro/, accessed on 30 July 2024) [64]. The theoretical isoelectronic point (pI) and molecular weight (Mw) were predicted online (https://web.expasy.org/compute_pi/, accessed on 30 July 2024) [65]. The signal peptide was predicted using the SingalP server (https://services.healthtech.dtu.dk/services/SignalP-6.0/, accessed on 30 July 2024) [66]. A phylogenetic tree of related protein sequences from PL7 and PL17 families was constructed using the neighbor-joining method with MEGA 11 [53]. Poly (beta-D-mannuronate) lyase (ABG42142.1) from the PL6 family was used as an outgroup in the phylogenetic tree. Multiple sequence alignment was performed among the characterized PL7 family alginate lyases using MEGA 11 and obtained using Jalview V2.10.5.

For further identification of strain HB236076, a genomic comparison was carried out using average nucleotide identity (ANI) and digital DNA–DNA hybridization (dDDH) with the close relative members. The ANI was calculated using the OrthoANIu algorithm by the EzGenome server (http://www.ezbiocloud.net/tools/ani, accessed on 30 July 2024) [67], and the dDDH value was estimated using the genome-to-genome distance calculator [68].

### 3.5. Detection of Alginate Lyase Activity and Enzymatic Properties

Strain HB236076 was propagated on the optimized liquid medium consisting of 5 g/L sodium alginate, 7.5 g/L trypsin, 25 g/L NaCl, 0.1 g/L MgSO_4_ · 7H_2_O, and pH 7.0 at 28 °C and 180 rpm for 12 h. Cells were removed by centrifugation at 10,000 rpm, 4 °C for 10 min, and then the alginate lyase activity of the supernatant was measured by the ultraviolet absorption method [69]. One unit of enzyme activity was defined as an increase of 0.1 in absorbance per min at 235 nm.

Unless otherwise stated, the following operating temperature of alginate lyase was carried out at 4 °C. Solid ammonium sulfate was added slowly to the cell-free crude enzyme solution to obtain 80% saturation and kept overnight. The resultant precipitate was centrifuged at 10,000 rpm for 15 min and then dissolved in 0.01 M phosphate-citrate buffer (pH 7.0). The enzyme solution was dialyzed four times in a dialysis bag (MWCO: 8000 Da) using the same buffer, changing the dialysis buffer every four hours. The dialyzed supernatant was used as partially purified alginase for the subsequent experiments.

The activities of alginate lyase at different temperatures (4 °C, 30–80 °C at 10 °C increments) and pH 7.0 were measured to investigate the effect of temperature on the enzyme activity. The enzyme was preincubated at different temperatures (4 °C, 30–80 °C) and pH 7.0 for 2 h to investigate the effect of temperature on alginate lyase stability. The enzyme activities at various pHs using Na_2_HPO_4_-citric acid (pH 3.0–8.0), Tris-HCl (pH 7.0–9.0), and glycine-NaOH (pH 9.0–10.0) buffers at 40 °C were measured to investigate the effect of pH value on the enzyme activity. The enzyme was preincubated in the buffers described above (pH 3.0–10.0) at 4 °C for 24 h to investigate the effect of pH value on alginate lyase stability. The highest activity mentioned above was taken as 100%. Under the optimum temperature and pH values, the enzyme activity was measured at 1 mM and 10 mM NaCl, KCl, MgCl_2_, MnSO_4_, ZnSO_4_, FeSO_4_, ethylenediamine tetraacetic acid (EDTA), and sodium dodecyl sulfate (SDS), respectively. Alginate lyase activity was measured as described above, with an untreated activity of 100%. All reactions were performed in triplicate.

### 3.6. Preparation and Detection of the Degradation Products of the Alginate Lyase

To investigate the degradation pattern of the alginate lyase, alginate degradation was performed with 15 g/L sodium alginate in 50 mM phosphate buffer (pH 7.0) as a substrate. The experiments were carried out under the conditions of 19.5 U/mL enzyme, pH 7.0, and 40 °C for 0–36 h. At intervals (3, 6, 24, and 36 h), aliquot samples (10 mL) were taken, boiled for 10 min to denature the enzymes, and then centrifuged at 12,000 rpm for 10 min to remove the undissolved substances.

The lysates were precipitated overnight with three times the volume of ethanol at 4 °C and then centrifugated at 12,000 rpm for 10 min. The supernatants were collected, lyophilized, and further redissolved in ultrapure water for the next tests. The TLC method was applied to examine the AOS with different DP produced by the alginate lyase. The degradation product compositions were analyzed by the TLC method on a silica gel high-performance TLC plate (Merck, Germany) with 1-butanol/formic acid/water (4:6:1 v:v:v) as a mobile solvent. Then, the plate was visualized by heating at 110 °C for 10 min after spraying with 10% (*v*/*v*) sulfuric acid in ethanol [70]. The guluronic acid sodium salt monomer, dimer and trimer (1 mg/mL) (Qingdao Bozhi Huili Biotech) were used as standards. In the ESI-MS analysis, the supernatant (2 µL) was loop-injected into the ESI-MS instrument (Bruker Compact, Bremen, Germany) after filtering by a 0.45 µm membrane. The oligosaccharides were detected in negative-ion mode using the following settings: end plate offset, 500 V, 131 nA; capillary, 4500 V, 2505 nA; nebulizer, 0.4 bar; dry gas, 4.0 L/min; dry temperature, 220 °C. 

### 3.7. Assays for Antimicrobial Activity 

Antimicrobial activity on seven pathogenic bacteria was determined by the agar diffusion method in this study. All the bacteria were incubated overnight at 37 °C in LB medium (Solarbio) for testing. While the autoclaved LB agar (Solarbio) was cooled to around 45 °C, the overnight culture was added and mixed to a cell density of approximately 10^6^ CFU/mL. After drilling wells (8 mm in diameter) on the inoculated LB plate, 50 μL oligosaccharide solution (1.91 mg/mL) filtered through 0.45 μm membrane were added to the wells. The diameter of the inhibition zone was measured after the inoculated LB plate was incubated at 37 °C for 24 h.

## 4. Conclusions

In this work, an alginate lyase-excreting marine bacterium, designated HB236076, was considered to represent a potential novel species of the genus *Vibrio* based on the phylogenetic and genetic data. The isolate presented two chromosome genomes with sizes of 3,007,948 bp and 874,895 bp. The CAZymes analysis result indicated that the isolate encoded 73 CAZymes, including nine alginate lyases in PL7 and PL17 families. The extracellular alginate lyase of strain HB236076 exhibited a wide range of temperature and pH activity and stability. The AOS of sodium alginate degraded by the extracellular alginate lyase mainly contained disaccharides and trisaccharides, which showed antimicrobial activity against several pathogenic bacteria. The results indicated that *Vibrio* sp. HB236076 was a good source of pH- and thermo-stable alginate lyase and oligosaccharide preparation.

## Figures and Tables

**Table 1:** Characteristics of the alginate lyases identified in the genome of strain HB236076.

Enzymes	Accession No.	PL Family	Length (aa)	MW (Dal)	Signal Protein		Sequence Comparison ^1^	
						PI	Sequence Identity (%)	Reference Alginate Lyase
Alg1	XDK25489.1	PL 7	279	30,235.57	Yes	3.90	61.9	GAA4099934.1
Alg2	XDK26069.1	PL 7	652	71,934.82	Yes	5.66	79.6	WP_231565442.1
Alg3	XDK26066.1	PL 7	343	38,020.27	Yes	6.17	76.0	WP_102470889.1
Alg4	XDK25655.1	PL 7	285	32,002.96	Yes	6.80	97.2	ABB36771.1
Alg5	XDK24838.1	PL 7	248	28,306.44	Yes	4.50	97.0	ABB36772.1
Alg6	XDK24840.1	PL 7	342	37,836.15	Yes	6.60	60.6	WP_334550621.1
Alg7	XDK24841.1	PL 7	302	33,260.67	Yes	4.51	47.9	WP_318074641.1
Alg8	XDK24833.1	PL 17	720	80,516.22	No	5.39	65.1	CAH6913425.1
Alg9	XDK24834.1	PL 17	720	80,465.18	No	5.68	63.2	WP_252024937.1

^1^: The reference sequences did not include the sequence from strain HB161653 we submitted.

## Data Availability

Data are contained within the article and Supplementary Materials.

## Supplementary Materials

The following are available online at https://www.mdpi.com/article/10.3390/md22090414/s1: Table S1: General genome features of *Vibrio* sp. HB236076; Table S2: ANI, dDDH, and 16S rRNA gene similarity values of strain HB236076 and the phylogenetically related taxa; Table S3: The polysaccharide-degrading enzymes predicted from the genome of *Vibrio* sp. HB236076; Figure S1: Gellation reaction of strain HB236076 observed on the plate covered by CaCl_2_ solution; Figure S2: Antibacterial activity testing plates.
