# Supplementary material for: Genome Analysis of a Potential Novel Vibrio Species Secreting pH- and Thermo-Stable Alginate Lyase and Its Application in Producing Alginate Oligosaccharides
Source: Mar Drugs. 2024 Sep 10;22(9):414. doi: 10.3390/md22090414 (PMC11433491; doi:10.3390/md22090414)
Supplement: Supplementary file 1 [file marinedrugs-22-00414-s001.zip › marinedrugs-3167792-supplementary.pdf]

marine drugs

Article

Genome Analysis of a Potential Novel *Vibrio* Species Secreting pH- and Thermo-Stable Alginate Lyase and Its Application in Producing Alginate Oligosaccharides

Ke Bao [1,2], Miao Yang [1,3], Qianhuan Sun [2], Kaishan Zhang [2,*] and Huiqin Huang [1,*]

1 Institute of Tropical Bioscience and Biotechnology, Hainan Institute for Tropical Agricultural Resources, Chinese Academy of Tropical Agricultural Sciences, Haikou 571101, China; 17326063163@163.com (K.B.); 2575128316@qq.com (M.Y.)
2 Hangzhou Watson Biotechnology Co., Ltd, Hangzhou 311400, China; sunqianhuan@watsonbiotech.com
3 College of Life Science and Technology, Huazhong Agricultural University, Wuhan 430070, China
* Correspondence: huazkskk@watsonbiotech.com (K.Z.); huanghuiqin@itbb.org.cn (H.H.)

Table S1. General genome features of *Vibrio* sp. HB236076.

**Category**	**HB236076**
GenBank No.	CP162601, CP162602
Isolation sample	*Sargassum* sp.
Contigs	2
Genome size (Mb)	3,882,843
Chromosome 1	3,007,948
Chromosome 2	874,895
G+C content (%)	46.47
Total genes predicted	3482
Protein-coding genes	3332
tRNA genes	116
rRNA genes	34
5S rRNA	12
16S rRNA	11
23S rRNA	11
CRISPR arrays	11

Table S2. ANI, dDDH, and 16S rRNA gene similarity values of strain HB236076 and the phylogenetically related taxa.

**Items (%)**	**1-2***	**1-3**	**1-4**	**1-5**	**1-6**
16S rRNA gene	98.4	98.1	98.0	98.0	97.4
ANI	71.1	70.8	71.9	71.9	70.8
dDDH	23.2	22.3	23.9	23.7	22.7

*Strains: 1, HB236076; 2, *V. maritimus* R-40493^T; 3, *V. variabilis* R-40492^T; 4, *V. japonicus* JCM 31412^T; 5, *V. sinaloensis* CAIM 797^T; 6,*V. halioticoli* NBRC 102217^T.

Table S3. The polysaccharide-degrading enzymes predicted from the genome of *Vibrio* sp. HB236076.

Catabolic Enzymes	Enzyme Family	No. of Enzymes
Alginate lyase	CBM32丨CBM32丨PL7	1
	PL7	6
	PL17	2
α-amylase	GH13	2
β-galactosidase	GH2	1
Glucosylceramidase	GH30	1
Peptidoglycan hydrolase	GH73	1
1,4-*α*-glucan-branching enzyme (GlgB)	CBM48丨GH13	1

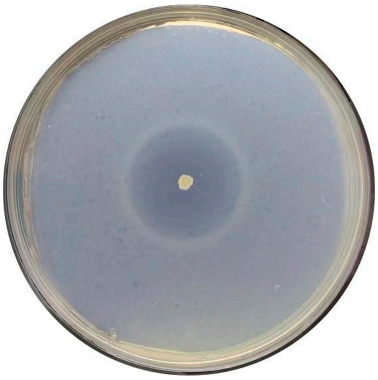

Figure S1. Gellation reaction of strain HB236076 observed on the plate covered by $CaCl_2$ solution.

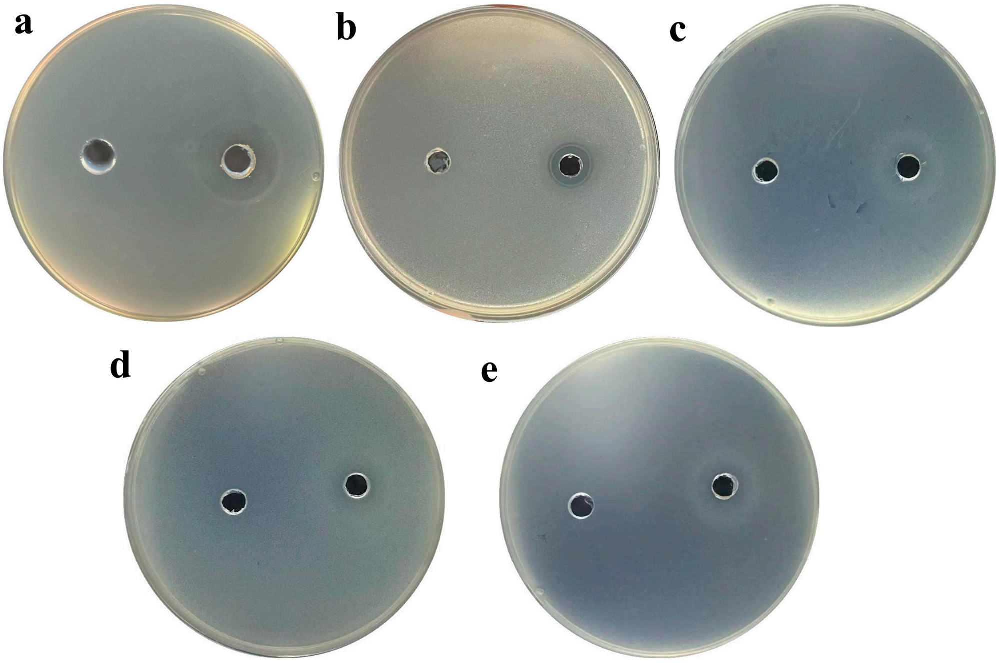

Figure S2. Antibacterial activity testing plates. (**a**) *Shigella dysenteriae*; (**b**) *Aeromonas hydrophila*; (**c**) *Staphylococcus aureus*; (**d**) *Streptococcus agalactiae*; (**e**) *Escherichia coli*. The left well was for water control, and the right well was for oligosaccharide sample.
